# Task-specific resistance training adaptations in older adults: comparing traditional and functional exercise interventions

**DOI:** 10.3389/fragi.2024.1335534

**Published:** 2024-04-30

**Authors:** Jason I. Pagan, Bethany A. Bradshaw, Brisilda Bejte, Jordan N. Hart, Vanjeliz Perez, Kevan S. Knowles, Jonathan P. Beausejour, Marc Luzadder, Reed Menger, Carlos Osorio, Kylie K. Harmon, William J. Hanney, Abigail T. Wilson, Jeffrey R. Stout, Matt S. Stock

**Affiliations:** ^1^ Institute of Exercise Physiology and Rehabilitation Science, University of Central Florida, Orlando, FL, United States; ^2^ Department of Exercise Science, Syracuse University, Syracuse, NY, United States

**Keywords:** sarcopenia, dynapenia, neuromuscular, aging, older adults, specificity

## Abstract

Muscle strength declines ∼3% per year after the age of 70. Resistance training guidelines for older adults are often based on free-weight and machine exercises, which may be inaccessible and lack carryover to activities of daily living. We tested the hypothesis that resistance training adaptations in older adults are task-specific. Thirty adults (8 males, 22 females; mean age = 71 years) were randomly assigned to participate in 6 weeks of supervised, high-intensity resistance training (twice per week) utilizing free-weight and machine exercises (traditional) *versus* functional activities that were overloaded with a weighted vest (functional). Participants were thoroughly familiarized with the exercises and testing prior to beginning the study. Major outcome measures included assessments of functional performance, five-repetition maximum strength, isometric knee extensor force, and quadriceps muscle size. Physical activity and nutrition were monitored. The study results demonstrate that the magnitude of improvement within a given outcome was largely dependent on group assignment, with greater improvements in gait speed and the timed-up-and-go in the functional group, but 2-3× greater five repetition maximum strength improvements for the trap bar deadlift, leg press, and leg extension following traditional resistance training. Both groups showed improvements in isometric knee extensor force and muscle size, suggesting that some aspects of the observed adaptations were generic, rather than specific. Overall, these novel findings suggest that, among older adults, 1) resistance training adaptations exhibit a high degree of task specificity and 2) significant improvements in functional outcomes can be achieved with the use of a weighted vest.

## Introduction

A significant percentage of the aging global population will lose a substantial amount of skeletal muscle mass, strength, and function, collectively known as sarcopenia (Cruz-Jentoft et al., 2014; Cruz-Jentoft et al., 2019; Mende et al., 2022). The concern of sarcopenia is increasingly magnified in industrialized societies in which adults are living into their 80s, 90s, and beyond, but with multiple co-morbidities and a sedentary lifestyle. The negative implications for older adults that gradually lose muscle mass and strength include limited mobility (Laukkanen et al., 1995), physical disability (Manini, 2011), and mortality (Newman et al., 2006). These complications result in an inability to perform activities of daily living, the loss of independence, and an overall reduction in quality of life (Papa et al., 2017; Fragala et al., 2019). Fortunately, resistance training serves as a potent nonpharmacological stimulus that can prevent or even reverse these detrimental effects (Westcott, 2012).

In older adults, substantial benefits from resistance training have been observed in ≤ 8 weeks, with training frequencies of only two sessions per week. Scanlon et al. (2014) reported that just 6 weeks of lower-body resistance training resulted in a 31.9% increase in knee extension one-repetition maximum (1RM) strength and a 31.5% increase in muscle quality (defined by their group as 1RM strength relative to dual-energy X-ray absorptiometry derived thigh lean mass). More recently, Herda and Nabavizadeh (2021) sought to compare the effects of only 6 weeks of progressive dumbbell *versus* elastic band resistance training on multiple measures of muscle function and mass in older adults. Their findings indicated that muscle strength improved in both groups, although small changes were observed for lean mass, suggesting that the underlying mechanisms for improved muscle quality may have been neural in nature. Older adults have also shown improvements in physical function measures following relatively short training interventions. In a study of older males >80 years of age, Kalapotharakos et al. (2010) concluded that 8 weeks of resistance training resulted in significant improvements in 3RM strength, 6-min walk distance, chair rise time, and timed-up-and-go (TUG) tests. Given the shorter duration of these investigations, it has been proposed that high-intensity (70%–85% of 1RM load) resistance training may inhibit neuromuscular aging by improving motor unit integrity, the rate of force development, and corticospinal excitability (Lavin et al., 2019). Importantly, these adaptations have been demonstrated even in the oldest old (Fiatarone et al., 1994; Basco, 2020).

Careful consideration of resistance training variables is crucial for facilitating favorable and specific adaptations in older adults. Contemporary, evidence-based recommendations for enhancing muscle function in older adults were recently described in a National Strength and Conditioning Association Position Statement authored by Fragala et al. (2019). Key elements to consider when designing effective resistance training programs for older adults include training volume, intensity, frequency, and exercise selection. For healthy older adults, one to three sets per muscle group with 8–15 repetitions per set for 2–3 days per week have been shown to be of sufficient volume and frequency to optimize training adaptations (Chodzko-Zajko et al., 2009; Fragala et al., 2019). In terms of training intensity, 70%–85% of the 1RM is commonly recommended for enhancing maximal strength, whereas lower training intensities may still prove useful in improving muscle mass (Fragala et al., 2019). However, for novices, frail older adults, and those with cardiovascular or metabolic disease, it may be beneficial to begin with lower intensities (e.g., 20%–30% 1RM) and gradually increase training intensity over time, regardless of the training goal (Chodzko-Zajko et al., 2009; Fragala et al., 2019). Generally, exercises that simultaneously stress multiple joints and muscle groups (e.g., deadlifts and leg presses) may elicit more favorable outcomes and have better carryover to functional tasks than single-joint exercises (e.g., knee extensions and biceps curls) (Chodzko-Zajko et al., 2009; Fragala et al., 2019).

Training specificity is also an important concept when considering improvements that occur in response to an exercise intervention. Specifically, the magnitude by which pretest-posttest improvements are observed in response to resistance training are reflective of how specific the training program is to the testing methods. This concept was illustrated in a classic 12-week study by Rutherford and Jones (1986). Their work showed that unilateral concentric knee extension training resulted in nearly a 200% increase in the loads used during training, but this resulted in only a 15%–20% increase in maximal isometric force. Thompson et al. (2015) examined changes in the rate of torque development for the knee extensors and vertical jump height in untrained males and females participating in 10 weeks of supervised barbell deadlift training. While significant improvements in the rate of torque development (18.8%–49.0%) and vertical jump height (7.4%) were observed, these improvements failed to reflect the nearly 100% increase in external loads that participants were able to use in their deadlift training. Further emphasizing the importance of specificity, Mattocks et al. (2017) compared improvements in 1RM strength between participants that performed a high-volume training program based on guidelines for maximizing strength *versus* a group that simply practiced 1RM testing. Their findings indicated that, for upper- and lower-body exercises, both groups observed similar improvements in 1RM strength, questioning the need to perform high-volume training if one’s goal is simply to improve maximal strength. While these concepts are becoming increasingly well-established in younger adults, less is known about the role of resistance training specificity in older adults. Studying this topic across the lifespan is important, as older adults may be hesitant to perform certain types of exercise programs without qualified supervision or lack access to appropriate equipment (Schutzer and Graves, 2004).

In 2019, the European Working Group on Sarcopenia in Older People (EWGSOP) published updated guidelines concerning the definition and diagnosis of older adults with sarcopenia (Cruz-Jentoft et al., 2019). Their report highlighted a variety of tests that should be used to assess aspects of lower-body muscle function in older adults. These include gait speed, TUG, chair stands, and the 4-m walk test, all of which are simple to perform and require minimal resources. In addition to the use of these tests to examine changes following clinical interventions, several have been proposed as tools for screening for sarcopenia (Cruz-Jentoft et al., 2019). However, while these tests may be valuable for assessment and screening purposes, it is not clear if practicing these tests repeatedly results in test-specific performance. Moreover, as resistance training guidelines tend to be based on machine and free-weight exercises (Fragala et al., 2019), standard training protocols may lack important specificity components. Therefore, using a 6-week randomized controlled trial, the purpose of the present study was to compare the effectiveness of a traditional machine and free-weight resistance training program based on consensus guidelines (Fragala et al., 2019) to a functional resistance training program specifically designed to improve performance on specific tests recommended for the diagnosis of sarcopenia in older adults (Cruz-Jentoft et al., 2019). We speculated that pretest-posttest improvements would be highly specific to group assignments. Specifically, we hypothesized that older adults engaged in machine and free-weight training would show significant improvements in 5RM strength in each of their assigned exercises, but smaller improvements in lower-body muscle function tests. Conversely, we theorized that older adults that were able to practice performing lower-body muscle function tests would show substantial improvements in those tests, but smaller changes in 5RM strength. To examine the extent to which changes are non-specific, we included a unilateral isometric knee extension maximal voluntary contraction (MVC) to assess general strength, in addition to using B-mode ultrasonography to examine changes in skeletal muscle mass. The findings from this study have important implications for exercise program design in older adults, particularly in situations where access to exercise equipment may be limited.

## Materials and methods

### Experimental design

**FIGURE 1:**
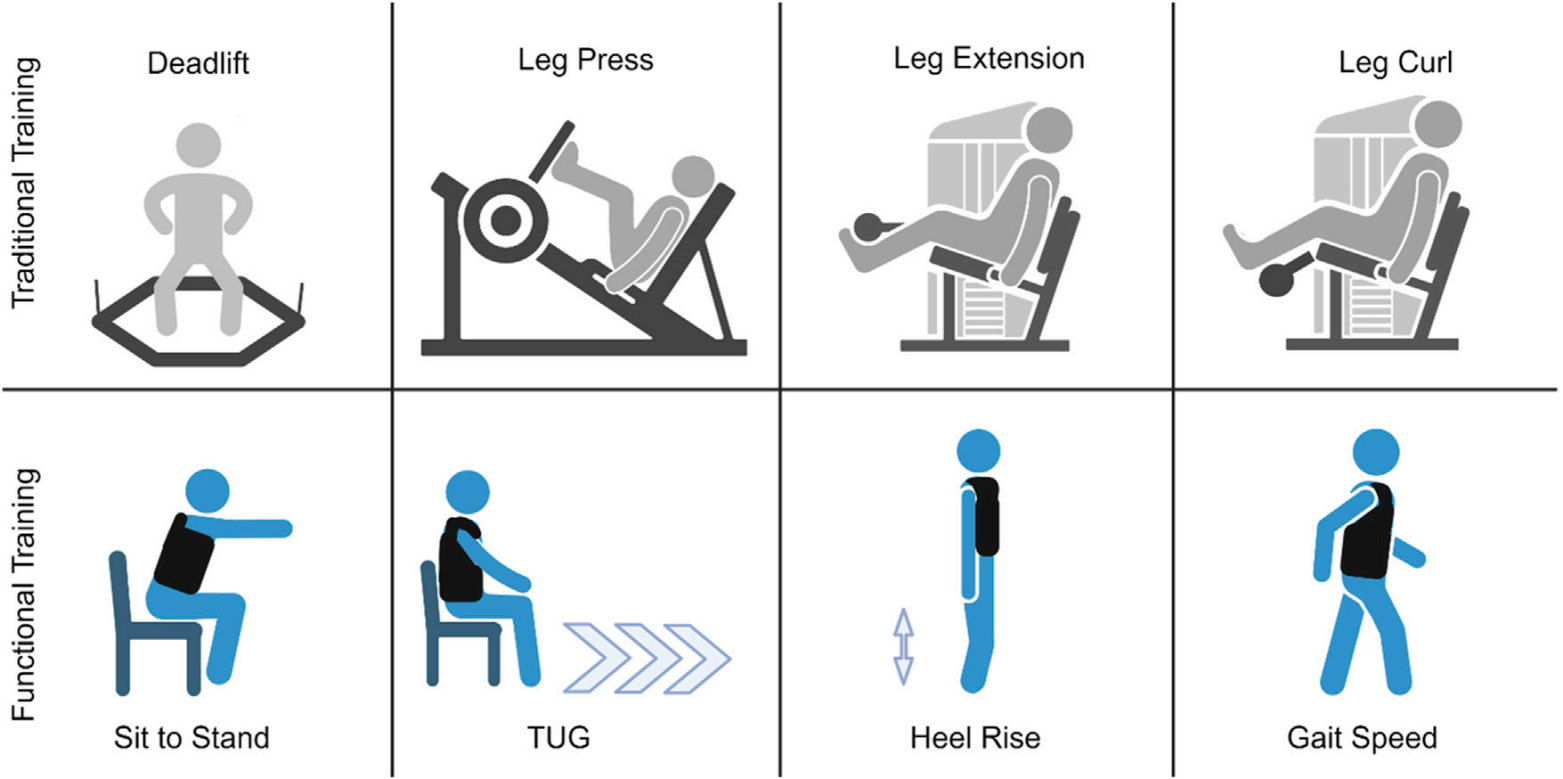
A schematic overview of the exercises and tests performed by the two groups.

The study utilized a 6-week, randomized pretest/posttest design. Participants were blocked on gender during enrollment to ensure a similar distribution of males and females in each group. Once enrolled, participants were randomly assigned into one of two groups: 1) traditional machine and free-weight resistance training and 2) functional resistance training (Figure 1). Both groups visited the University of Central Florida (UCF) for training twice per week at the same time of day (± 1 hour), with visits ≥48 h apart. Participants became acquainted with the exercises and testing protocol during an initial familiarization visit to minimize learning effects. Investigators demonstrated each test prior to testing. Following the initial familiarization visit, participants engaged in two separate pretesting visits. The data from these visits were used to calculate study-specific test-retest reliability data (i.e., intraclass correlation coefficients [ICCs], standard error of measurement [SEM], minimal important difference [MID]), which were used to quantify the effectiveness of the training programs on a participant-by-participant basis. A comprehensive testing battery was used to evaluate pre-post changes and included ultrasound assessments of the vastus lateralis (VL) and rectus femoris (RF), unilateral knee extension MVC force, functional performance tests, and 5RM strength. Passive and effortless tests were performed prior to tests which were more fatiguing. The order in which tests have been described below corresponded to the order in which they were performed. Each participant completed the tests in the same order each testing session. Testing sessions were conducted at the same time of day (± 1 hour) to minimize diurnal effects. All resistance training sessions (12 total sessions) were supervised by investigators at UCF to verify proper completion of each session. Nutrition and physical activity levels outside of the laboratory were monitored at the beginning and end of the study with 3-day food logs and accelerometers, respectively. Given the possibility of low protein diets among older adults (Deutz et al., 2014; Norton et al., 2016), we provided each participant with a ready-to-drink protein supplement (30 g of protein each, 60 g of protein total; Nutrition Plan^®^, Fairlife^®^, LLC, Chicago, IL, USA) before and after each training session to support muscle anabolism.

### Participants

Forty-three older adults (14 males, 29 females; mean age = 71 years) were enrolled, with a final sample size of 30 participants (8 males, 22 females; mean ± SD age = 71 ± 5 years, height = 164.5 ± 8.1 cm; mass = 72.1 ± 14.7 kg; body mass index = 26.5 ± 4.0 kg/m^2^) completing all study requirements. Recruitment was conducted through the community and the UCF Learning Institute for Elders group via flyers, word of mouth, social media, and a link to study information on the UCF College of Health Professions and Sciences research page. Prior to study enrollment, an initial screening was conducted to determine if interested individuals qualified for participation. Exclusion criteria included history of cancer, neuromuscular disease, metabolic disease, experience of myocardial infarction or lower-body surgery within the past year, the use of assistive walking aids, lactose-intolerance (due to the dairy-based protein shakes), physician mandated contraindication to exercise based on subject reporting and answering “YES” to any of the questions on the Physical Activity Readiness Questionnaire. Participants taking prescription medications were reviewed on a case-by-case basis; however, any prescription medications that caused muscle weakness were considered exclusionary. Finally, participants were required to have refrained from lower-body resistance training 6 months prior to study participation. All participants read and signed an informed consent document detailing study risks and procedures. All study procedures were approved by the University of Central Florida Institutional Review Board (IRB #STUDY00004684). Participants were compensated in the form of virtual Amazon gift cards ($100) disbursed via email for their time and transportation considerations. If participants were only able to complete the pretest visits but failed to complete the 6 weeks of training, they were compensated $25.

### Assessment of sarcopenia

Each participant completed the SARC-F questionnaire prior to testing. The SARC-F is a five-item questionnaire that can predict/screen individuals that are at risk of sarcopenia (Malmstrom et al., 2016). The five components of the SARC-F include: strength, assistance walking, rise from chair, climb stairs, and falls. Scoring for the SARC-F is based on a 0–10 scale (0 = best; 10 = worst) with each component being worth between 0 and 2 points (0 = no difficulty; 1 = little to some difficulty; 2 = lots of difficulty/require aid). A score equal to or greater than four is predictive of sarcopenia and poor outcomes. The SARC-F was used in this study for descriptive purposes.

In addition to the SARC-F, participants performed maximal grip strength testing to evaluate the presence of sarcopenia. During the familiarization session and two pretest visits, grip strength was examined for both hands. During each of the three visits, each participant performed six, 5-s maximal grip strength tests with each hand (three left and three right), but in alternating order (i.e., right, left, right, etc.). A digital hand grip dynamometer (Jamar Technologies, Patterson Medical, Warrenville, IL) was used, and participants were seated with their elbow at 90° flexion and wrist in a neutral position. A 1-min rest was provided between attempts. The single highest attempt (kg) across all visits, irrespective of hand dominance, was used for analysis. Cut points of <27 kg and <16 kg for males and females, respectively, were used to determine sarcopenic status (Cruz-Jentoft et al., 2019).

### Assessment of skeletal muscle size

A portable B-mode imaging device (GE Logiq e BT12, GE Healthcare, Milwaukee, WI, USA) and a multi-frequency linear-array probe (12 L-RS, 5 – 13 MHz, 38.4-mm field of view, GE Healthcare, Milwaukee, WI, USA) were used to take ultrasonography images of the VL and RF to assess muscle size prior to testing battery. Participants were first instructed to lie on their left side of the body with their legs on top of one another, with their knee joint positioned at an angle of 15°, on a treatment table to capture ultrasonography images of the VL. Participants were then instructed to lie down in a supine position on a treatment table and ultrasonography images of the RF were captured. Ultrasonography images of both muscles were captured in the transverse and sagittal plane using the panoramic function and B-mode function. The same investigator captured all ultrasonography images. The images were then analyzed using ImageJ (ImageJ, version 1.51, NIH, Bethesda, MD, USA). The polygon function was used to quantify muscle cross-sectional area (CSA) for the VL and RF.

### Maximal isometric force

A custom-made chair with a load cell (Interface, Inc., Force Measurement Solutions, SSMF Fatigue Rated S-type Load Cell, Scottsdale, AZ, USA) attached to the bottom right leg was used to assess maximal voluntary contraction (MVC) force of the dominant knee extensors. The back pad of the seat was adjusted to ensure participants were upright and the knee was aligned with the edge of the chair. A seat belt was fastened across the waist to reduce unnecessary movement from the hips and the right ankle was secured with an ankle strap to keep the right leg stationary. Prior to maximal testing, participants performed a submaximal warm-up of three 10-s contractions at 50% of their perceived maximal torque. Following the warm-up protocol, maximal isometric strength of the dominant knee extensors was assessed during 3 MVCs lasting 5 s each with 3-min rest between each contraction. Participants received verbal encouragement and were instructed to kick out “as hard and fast as possible”.

### Gait speed test

The 4-m gait speed test was used to assess physical function and mobility. Both the starting line and 4-m finish line were marked on the floor with masking tape. Participants began by walking 4 m from the starting point in a straight line until passing a cone at the end of the course (Bohannon and Wang, 2019). After an initial practice trial to ensure comfort with and comprehension of the procedures, participants performed an additional six timed trials: three at comfortable gait speed, followed by three at a fast gait speed. Timed trials were recorded in seconds with the use of timing gates (THE WITTY SYSTEM, Microgate, Mahopac, NY, USA) placed at the starting line and 4-m line. The times were then averaged from each trial and converted to meters per second for analysis.

### TUG

To assess balance and functionality (Mathias et al., 1986; Podsiadlo and Richardson, 1991), the timed-up-and-go (TUG) test was performed. The TUG began with participants standing up from a chair and walking along a marked line on the floor for a short distance (3 m), turning around to return to the chair, and sitting down on the chair. Timing began the moment the participant stood up from the chair and ended once they returned to the chair. After an initial practice trial, participants completed three timed trials at a comfortable walking pace. Timed trials were recorded in seconds with the use of a digital stopwatch (A601X-Pro Survivor Stopwatch, ACCUSPLIT, Pleasanton, CA, USA).

### Sit-to-stand chair test

The sit-to-stand chair stand test was used to assess lower body strength (Bohannon et al., 2010). At the start of the test, participants sat on a chair with their arms crossed. They were instructed to not sit back in the chair, ensuring that their back did not rest on the back pad of the chair. Participants were then instructed to stand up from the chair and sit back down for a total of five repetitions, while keeping their arms crossed across their chest and legs shoulder-width apart. Participants were instructed to not touch the back of the chair throughout the set. Time to complete five repetitions from the first stance to final sit was recorded in seconds with a digital stopwatch (A601X-Pro Survivor Stopwatch, ACCUSPLIT, Pleasanton, CA, USA).

### Heel-rise test

The heel-rise test was conducted to assess strength of the plantar flexors based on the recommendations of Lunsford and Perry (1995). The test began with participants standing upright near a wall, with their hands touching the wall for balance. They were then instructed to raise and lower the heel of their dominant foot while balancing on the ball of their foot and keeping the knee fully extended. Participants completed as many repetitions as possible. Testing was considered either complete or terminated if the participant leaned too far forward, the dominant knee became excessively flexed, or range-of-motion of the plantar flexion decreased >50% (Komforti et al., 2021).

### 5RM strength

To assess lower body muscular strength, five-repetition maximum testing (5RM) testing was conducted in the following order: trap-bar deadlift, leg press, leg extension, and leg curl. The 5RM testing protocol was adapted from Sheppard and Triplett (2016) and Stout et al. (2013) to determine maximal 5RM strength in the prescribed exercises. Briefly, participants completed two warm-up sets with a very light load for 5–10 repetitions, followed by 3–5 min rest between each set. The load was then increased an additional 10%–20% and participants were instructed to attempt to complete five repetitions. Participants that were able to successfully complete first 5RM attempt with proper technique were given 3–5 min rest before performing another 5RM attempt with the load increased an additional 4–8 kg. The 5RM test was completed once the participant was able to achieve a true 5RM, with proper form, within five attempts. If a participant had failed a 5RM attempt, the load from the previous attempt was recorded as the 5RM. Results from the 5RM test were used to estimate training loads for participants in the traditional resistance training group.

### Traditional machine and free-weight resistance training

At the start of each training session, warm-up sets were performed for the prescribed exercises prior to working sets. Participants that were randomly assigned to the traditional machine and free-weight group completed the following exercises in order: trap-bar deadlift, leg press, leg extension, and leg curl twice per week for 6 weeks. Each exercise was completed with all repetitions performed to failure for three sets at 85% of the estimated 1RM with 3 min of rest between sets and each exercise. Participants were instructed to perform each exercise with a full range of motion and were given the verbal cue “go as quickly as possible” on the concentric action of each exercise to ensure maximal velocity for the development of strength and power. Progressive overload was ensured by adding weight to each exercise throughout the study. Training loads were adjusted on a set-by-set basis. As 85% of the estimated 1RM corresponds roughly a 6RM load (Sheppard and Triplett, 2016) the goal for each set was for the participants to perform between three to nine repetitions for each set. For example, if a participant could only perform two repetitions for a given set, weight was removed prior to the next set. Conversely, performing ten repetitions for a given set would result in more weight being added for the next set. This set-by-set approach has proven successful for inducing rapid gain in strength in previous short-term studies (DeFreitas et al., 2011; Stock et al., 2017).

### Functional resistance training

Participants randomly assigned to the functional resistance training group completed modified variations of functional physical performance tests with a weighted vest (Rogue Fitness, Condor Sentry Plate Carrier, Toledo, OH, USA) in the following order: chair stands, TUG, heel rises, and 4-m gait speed twice per week for 6 weeks. Participants performed each exercise for five repetitions for three sets with ≤1 min rest between sets. Each movement was performed with full range of motion along with a verbal cue to “go as quickly as possible” on every exercise. Study investigators were present and served as spotters for every training session for both groups to monitor participant safety, ensure that participants were performing exercises with proper form, and give verbal encouragement. For the loading of the weighted vest, females started with 2 kg, and males started with 5 kg for their first training session. To induce progressive overload, total load of the vest increased either 2 kg or 5 kg at each training visit depending on how a participant performed at their previous session. If a participant was able to undergo a training session while maintaining the required intensity and adequately recover with the prescribed training load, we would increase 5 kg for the following session. If a participant was struggling to maintain intensity and somewhat recover with the prescribed training load, we would increase 2 kg for the following session. In the event a participant could not complete the required volume, along with displaying poor exercise form, with the prescribed training load, the load from their previous training session would be used.

### Physical activity monitoring

To assess whether habitual activity differed after the assigned interventions, daily physical activity for each participant was objectively measured at the start and end of the study via ActiGraph GT×9 Link (ActiGraph, Pensacola, FL, USA) accelerometers. The accelerometers were programmed to record acceleration in 60 s periods with low frequency extension (Kim et al., 2015). Participants were instructed to wear the accelerometers on the right side of the waist, attached via an elastic belt. The accelerometers were worn during all waking hours (except water-based activities) for seven consecutive days for a duration of 1 week. A valid day was considered as having ≥10 h of monitor wear time (Troiano, 2007). All data were processed using ActiLife version 6.13.3 software. All data points were entered into a wear-time validation program to determine if participants met the required amount of wear time during the monitoring period. The validation program also identified any periods of non-wear time, defined as ≥ 60 min of zero counts, which were then excluded from the analysis (Troiano, 2007). To determine physical activity levels, assessment of counts per minute (CPM) was utilized (Troiano, 2007). Activity counts were averaged into 60-s epochs or counts per minute (CPM), and then categorized into minutes of activity intensity using a validated algorithm (Troiano, 2007) The algorithm established the following CPM cut-off points: sedentary = 0 – 99 CPM, light = 100 – 2019 CPM, moderate = 2020 – 5998 CPM, and vigorous ≥5999 CPM. Minutes spent in each intensity level were then averaged across each day participant wore the accelerometer. These procedures were followed for both the baseline and post-intervention monitoring periods.

### Dietary analyses

Each participant was given a food log during the first week of training and the last week of training that required them to track all food and beverages consumed. They were asked to record a total of 6 days (3 days for the first week of training and 3 days for the last week of training). They were instructed to pick two weekdays and one weekend day to record their food and beverage intake. Participants were instructed to log every item as specifically as possible with item brand names, method of preparation, and measurement of food/beverage quantities if feasible. Participants were informed to keep their diet and caloric intake consistent throughout the study. Investigators also requested that participants be consistent with caffeine consumption. Investigators reminded the participants often about logging in their food logs during their training sessions. Food logs were collected on training session visits. The ready-to-drink protein shakes given before and after training sessions were not included for dietary analysis since participants were asked to not record them in their food logs. Total caloric intake, protein, carbohydrates, and fats were analyzed from each food log using an online software (MyFitnessPal, Inc., San Francisco, CA, USA). Changes in the consumption of these nutrients were evaluated (first week of training *versus* last week of training).

### Statistical analyses

This study’s statistical analyses involved multiple approaches. First, data from the two pretest visits were used to calculate test-retest reliability statistics using the guidelines described by Weir (2005). Specifically, for each dependent variable, the ICC, SEM (in absolute units), and SEM (as a percentage of the grand mean) were calculated, in addition to paired samples *t*-test and Cohen’s *d* effect sizes. Minimal important difference values (MID) were determined for both groups to consider the important change needed by multiplying the pooled SD from baseline of both groups with 0.5 (Norman et al., 2003).

Before analyzing any between-group differences, we first evaluated the assumption of normality for the data. Specifically, we conducted Shapiro-Wilk tests to assess normality. We also examined the skewness and kurtosis of the data to further evaluate the normality assumption. For each dependent variable, a two-way (time × group) mixed factorial analysis of variance (ANOVA) was used to determine statistically significant differences. In the event of a significant time × group interaction or significant main effects for time or group, Bonferroni-corrected *post hoc* pairwise comparisons were performed. Partial eta squared statistics (η_p_^2^) were used as a measure of the effect size for each ANOVA, with values of 0.01, 0.06, and 0.14 representing small, medium, and large effects, respectively (Cohen, 1988). An alpha level of 0.05 was used to determine statistical significance. In addition, we evaluated change scores on an individual participant basis to determine the number participants that showed changes exceeding the MID. Table 1 displays the results from our test-retest reliability analysis. JASP software (version 0.18.3, University of Amsterdam, Amsterdam, Netherlands) was used to conduct all ANOVAs, whereas a custom Excel spreadsheet (Microsoft Corporation, Redmond, WA, USA) was used to conduct all reliability analyses.

**TABLE 1:** Test-retest statistics for each of the dependent variables in this study. The pretests were performed following test familiarization. The SEM and MID are in the same units of measurement as the given dependent variable. VL = vastus lateralis; RF = rectus femoris; MVC = maximal voluntary contraction; TUG = timed up and go; 5RM = 5 repetition-maximum; ICC = intraclass correlation coefficient; SEM = standard error of measurement; MD = minimal difference needed to be considered real.

Dependent variable	Pretest #1	Pretest #2	*P*	*d*	ICC (3,1)	SEM	SEM (%)	MID
VL CSA (cm^2^)	16.5 ± 6.25	16.03 ± 5.198	0.248	0.193	0.927	1.55	9.55	2.77
RF CSA (cm^2^)	4.55 ± 1.68	4.34 ± 1.71	0.087	0.289	0.903	0.527	11.8	0.84
MVC Force (Newtons)	424.9 ± 151.3	430.5 ± 154.1	0.405	0.139	0.965	28.5	6.65	75.84
Gait Speed Normal (m×s^-1^)	1.29 ± 0.161	1.33 ± 0.159	0.018	0.408	0.812	0.069	5.31	0.080
Gait Speed Fast (m×s^-1^)	1.84 ± 0.322	1.88 ± 0.321	0.0075	0.302	0.945	0.075	4.05	0.16
TUG (sec)	8.69 ± 1.20	8.38 ± 1.21	0.004	0.511	0.876	0.426	4.99	0.60
Sit-to-Stand (sec)	11.8 ± 3.31	11.9 ± 2.82	0.902	0.020	0.397	2.39	20.2	1.53
Heel-Rise (reps)	22.1 ± 6.59	23.0 ± 7.87	0.251	0.192	0.807	3.19	14.1	3.62
Trap-Bar Deadlift 5RM (kg)	53.0 ± 18.9	57.0 ± 19.3	**0.002**	0.539	0.926	5.21	9.47	9.54
Leg Press 5RM (kg)	62.8 ± 33.5	73.0 ± 40.1	**<.001**	0.643	0.907	11.3	16.6	18.54
Leg Extension 5RM (kg)	28.9 ± 13.5	32.9 ± 14.2	**<.001**	0.787	0.931	3.62	11.7	6.94
Leg Curl 5RM (kg)	18.2 ± 8.91	19.5 ± 9.73	**0.008**	0.465	0.956	1.96	10.4	4.64

Bold font represents a significant increase from pretest #1 vs. pretest #2.

As a final exploratory aim, we examined Pearson *r* correlations between change scores in the primary dependent variables for the entire sample. Correlation coefficients were interpreted using established guidelines, with *r* values of 0.10–0.29 considered small, 0.30 to 0.49 medium, and 0.50 to 1.0 large in magnitude (Cohen, 1988). The JASP statistical software (version 0.18.3, University of Amsterdam, Netherlands) was used to generate heatmap visualizations depicting the results.

## Results

### Participants and descriptive statistics

**FIGURE 2:**
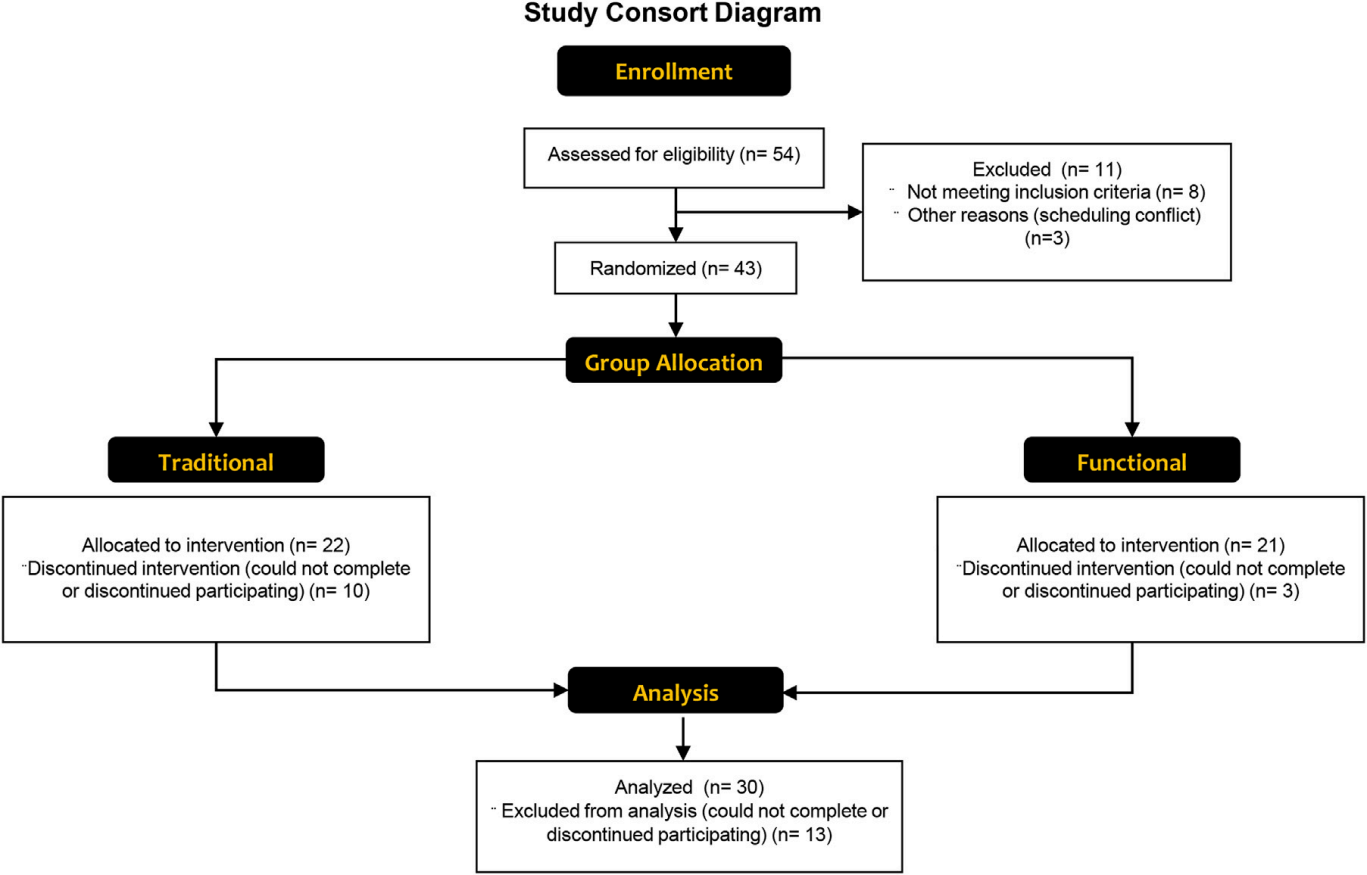
A CONSORT flow diagram showing the enrollment, group allocation, and analysis of the study participants. Reasons for withdrawal were unrelated to the study, and included unexpected difficulties with scheduling, musculoskeletal injury during accidents at home, and illness.

**FIGURE 3:**
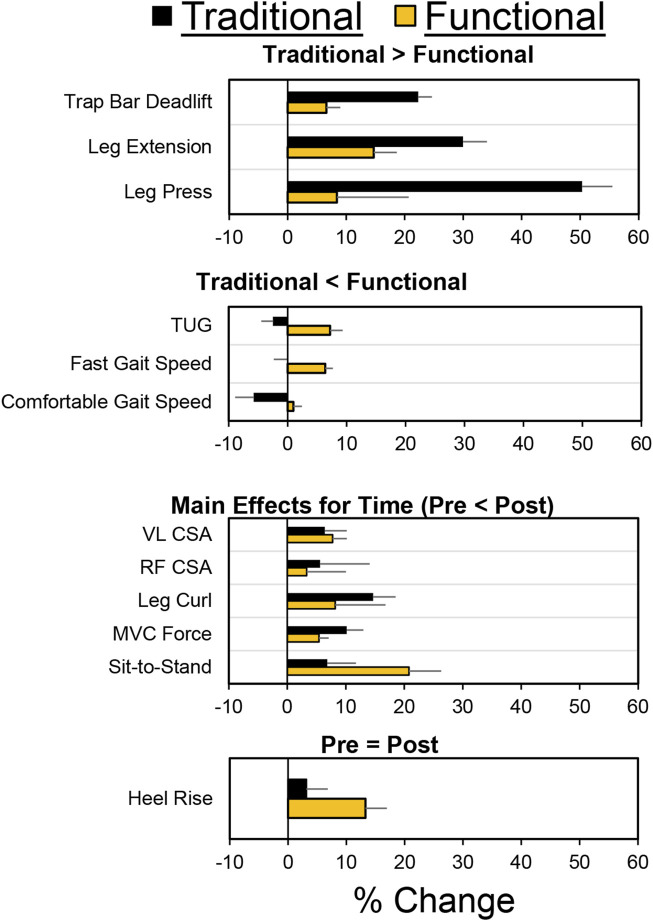
Mean pretest/posttest percentage changes for both groups and each dependent variable. For the purposes of this figure, data has been organized based on four types of findings: 1) Traditional > Functional; 2) Traditional < Functional; 3) Similar improvements in both groups (i.e., main effect for time); 4) Small changes for both groups (Pre = Post). Negative values represent worse performance on the posttest. Error bars represent standard error of the mean.

As shown in Figure 2 54 older adults were assessed for their eligibility to participate. Thirty older adults completed the study, with 18 and 12 in the functional and traditional groups, respectively. Three out of the 30 participants were considered at risk for sarcopenia (2 in traditional, one in functional) based on their recorded responses from the SARC-F questionnaire (≥ total score of 4), whereas 4 (2 in traditional, two in functional) were considered at risk for sarcopenia based on grip strength. There were no differences between groups at baseline for any of the dependent variables (*p* > 0.05). All 30 participants attended 100% of their scheduled visits as requested. A comprehensive overview of mean percent changes within each group for each dependent variable has been shown in Figure 3.

The Shapiro-Wilk test revealed that the following dependent variables exhibited non-normal distributions (*p* < .05): TUG, Sit-to-Stand, Trap Bar Deadlift 5RM, and Leg Press 5RM. This non-normality was primarily due to positive skewness, with a small number of participants demonstrating exceptionally slow TUG and Sit-to-Stand times or high Trap Bar Deadlift and Leg Press 5RM strength. To address the non-normal data, we explored using aligned-rank transforms for nonparametric factorial ANOVA, as described by Wobbrock et al. (2011). However, the results from these nonparametric analyses did not meaningfully differ from the findings of the conventional parametric ANOVAs. Therefore, we elected to maintain a consistent analytical approach across both the parametric and nonparametric datasets.

### 4-m gait speed—Comfortable

**FIGURE 4:**
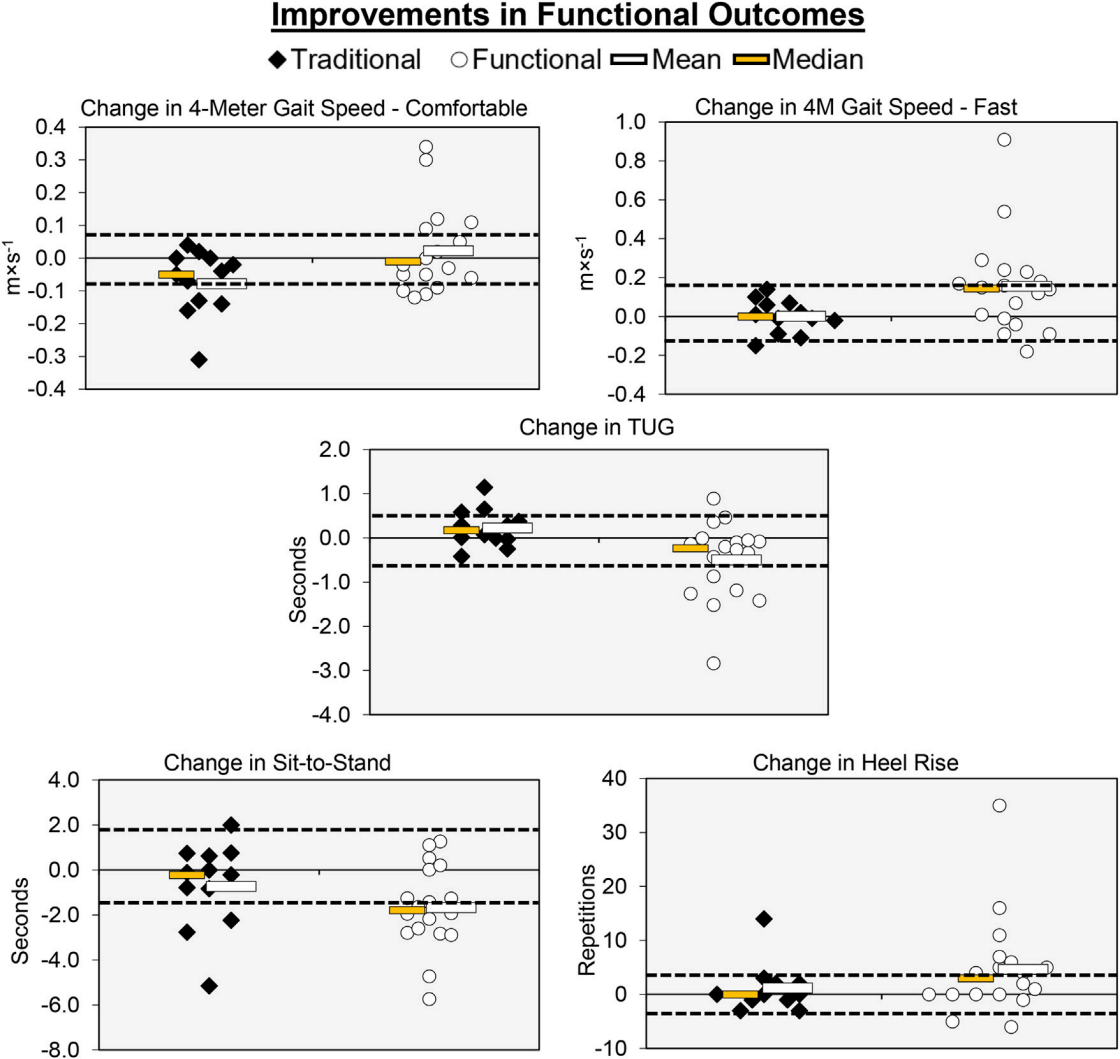
Individual participant, mean, and median changes for five of the functional performance tests for both training groups. The dotted red lines represent the MID.

Individual participant and mean changes for the functional outcomes have been displayed in Figure 4. The pretest and posttest mean ± SD comfortable gait speed values (m·s^-1^) for the traditional group were 1.31 ± 0.18 and 1.24 ± 0.14, respectively. These corresponding values for the functional group were 1.34 ± 0.13 and 1.36 ± 0.17. The results from the two-way mixed factorial ANOVA indicated that there was a significant time × group interaction (*F* = 4.384, *p* = 0.045, η_p_^2^ = 0.135). The results from the Bonferroni corrected post-hoc comparisons revealed a modest decrease in comfortable gait speed for the traditional group (*d* = 0.459) and a trivial/small improvement in comfortable gait speed for the functional group (*d* = 0.144), both of which were not statistically significant (*p* = 0.294 and *p* = 1.000, respectively). The number of participants that exceeded the MID of 0.080 m×s^-1^ in the traditional and functional groups was 4 (33.3%) and 9 (50.0%), respectively.

### 4-m gait speed—Fast

The pretest and posttest mean ± SD fast gait speed values (m×s^-1^) for the traditional group were 1.87 ± 0.29 and 1.87 ± 0.28, respectively. These corresponding values for the functional group were 1.85 ± 0.28 and 2.00 ± 0.42. The results from the two-way mixed factorial ANOVA indicated that there was a significant time × group interaction (*F* = 4.238, *p* = 0.049, η_p_^2^ = 0.131). The results from the Bonferroni corrected post-hoc comparisons demonstrated that there was a significant improvement in fast gait speed for the functional group (*p* = 0.018, *d* = 0.474). Fast gait speed did not improve for the traditional group (*p* = 1.000, *d* = 0.073). The number of participants that exceeded the MID of 0.16 m×s^-1^ in the in the functional group was 9 (50.0%). None of the participants in the traditional group exceeded the MID.

### TUG

The pretest and posttest mean ± SD TUG values (seconds) for the traditional group were 8.36 ± 1.12 and 8.59 ± 1.14, respectively. These corresponding values for the functional group were 8.50 ± 1.26 and 8.00 ± 1.25. The results from the two-way mixed factorial ANOVA indicated that there was a significant time × group interaction (*F* = 6.902, *p* = 0.014, η_p_^2^ = 0.198). The results from the Bonferroni corrected post-hoc comparisons demonstrated that participants in the functional group performed the TUG faster following training (*p* = 0.045, *d* = 0.415). The traditional group showed no improvement in the TUG (*p* = 1.000, *d* = 0.112). The number of participants that exceeded the MID of 0.60 s in the traditional and functional groups was 2 (16.7%) and 7 (38.9%), respectively.

### Sit-to-Stand

The pretest and posttest mean ± SD sit-to-stand values (seconds) for the traditional group were 11.64 ± 2.47 and 10.95 ± 1.69, respectively. These corresponding values for the functional group were 11.91 ± 2.92 and 10.24 ± 3.51. The results from the two-way mixed factorial ANOVA indicated that there was no significant time × group interaction (*F* = 1.927, *p* = 0.176, η_p_^2^ = 0.064), as well as no main effect for group (*F* = 0.046, *p* = 0.832, ή^2^ = 0.002). There was, however, a significant main effect for time (*F* = 11.249, *p* = 0.002, ή^2^ = 0.287). The results from the Bonferroni corrected pairwise comparisons indicated that, when collapsed across group, Sit-to-Stand times decreased from 11.8 ± 0.5 to 10.6 ± 0.6 s. The number of participants that exceeded the MID of 1.53 s in traditional and functional groups was 3 (25.0%) and 10 (55.6%), respectively.

### Heel-rise

The pretest and posttest mean ± SD heel-rise values (repetitions) for the traditional group were 22.67 ± 7.84 and 23.83 ± 8.77, respectively. These corresponding values for the functional group were 23.94 ± 8.92 and 28.61 ± 10.79. The results from the two-way mixed factorial ANOVA indicated that there was no significant time × group interaction (*F* = 1.484, *p* = 0.233, η_p_^2^ = 0.050), as well as no main effect for group (*F* = 0.919, *p* = 0.346, ή^2^ = 0.032) or time (*F* = 4.123, *p* = 0.052, η_p_^2^ = 0.128). The number of participants that exceeded the MID of 3.62 reps in the traditional and functional groups was 1 (8.33%) and 11 (61.1%), respectively.

### MVC force

**FIGURE 5:**
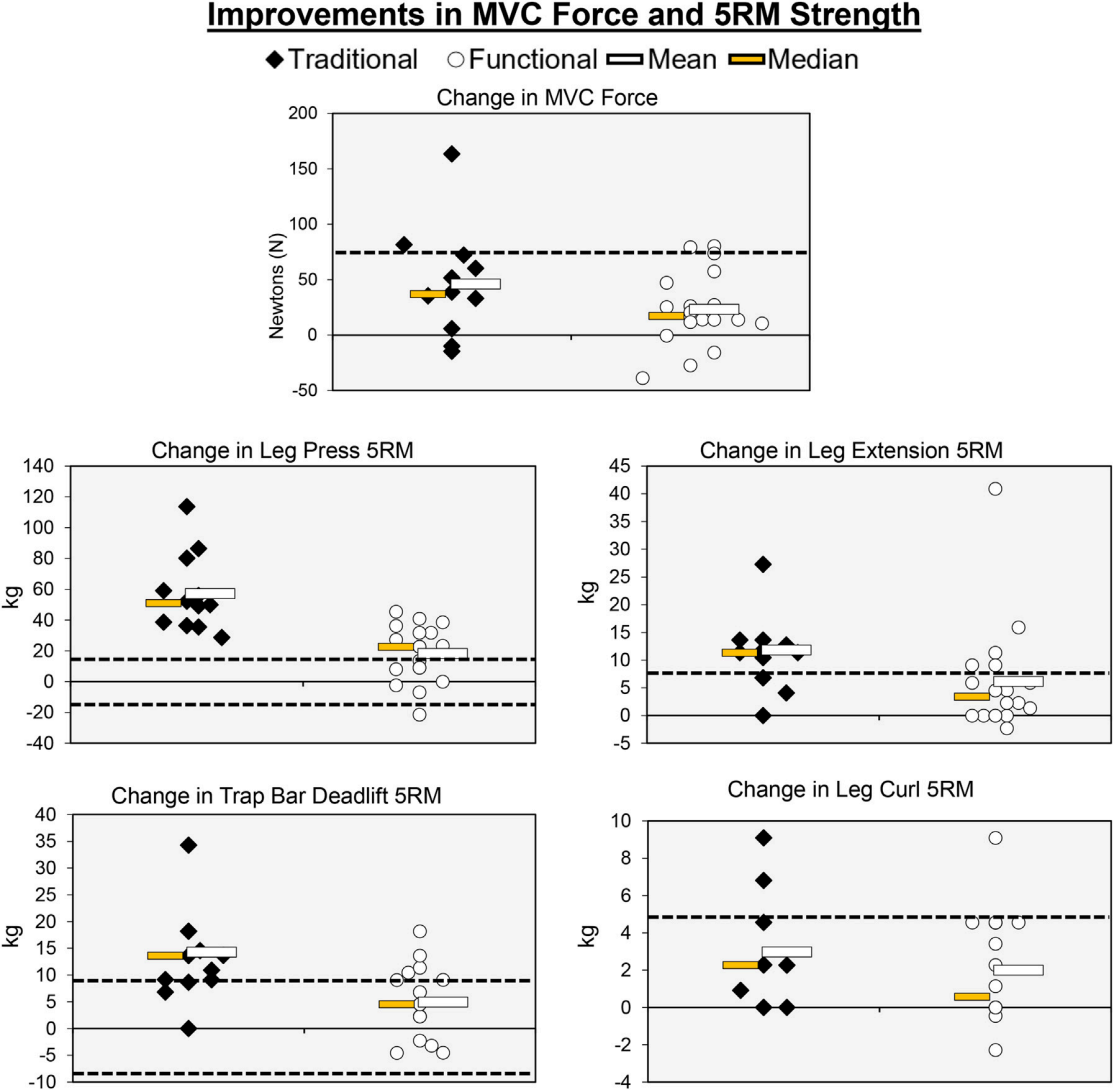
Individual participant, mean, and median changes for the maximal strength tests for both training groups. The dotted red lines represent the MID.

Individual participant and mean changes for the functional outcomes have been displayed in Figure 5. The pretest and posttest mean ± SD MVC force values (N) for the traditional group were 385.33 ± 124.35 and 431.37 ± 148.47, respectively. These corresponding values for the functional group were 426.81 ± 174.54 and 450.07 ± 175.63. The results from the two-way mixed factorial ANOVA indicated that there was no significant time × group interaction (*F* = 2.344, *p* = 0.137, η_p_^2^ = 0.077), as well as no main effect for group (*F* = 0.255, *p* = 0.618, η_p_^2^ = 0.009). There was, however, a significant main effect for time (*F* = 21.721, *p* < 0.001, η_p_^2^ = 0.437). The results from the Bonferroni corrected pairwise comparison indicated that, when collapsed across group, MVC force increased from 406.1 ± 29.2 to 440 ± 30.8 N. The number of participants that exceeded the MID of 75.84 N in the traditional group and functional group was 2 (16.7%) and 2 (11.1%), respectively.

### Trap bar deadlift 5RM

The pretest and posttest mean ± SD trap bar deadlift 5RM values (kg) for the traditional group were 52.50 ± 19.63 and 67.36 ± 24.29, respectively. These corresponding values for the functional group were 59.29 ± 21.42 and 64.24 ± 24.01. The results from the two-way mixed factorial ANOVA indicated that there was a significant time × group interaction (*F* = 19.018, *p* < 0.001, η_p_^2^ = 0.349). The results from the Bonferroni corrected pairwise comparisons showed significant increases in trap bar deadlift 5RM for the traditional group (*p* < 0.001) and functional group (*p* = 0.029). However, the magnitude of improvement was ×3 greater for the traditional group (*d* = 0.661 *versus d* = 0.220). The number of participants that exceeded the MID of 9.54 kg in the traditional and functional groups was 8 (66.7%) and 4 (22.2%), respectively.

### Leg press 5RM

The pretest and posttest mean ± SD leg press 5RM values (kg) for the traditional group were 67.82 ± 49.60 and 125.002 ± 54.92, respectively. These corresponding values for the functional group were 78.31 ± 39.27 and 96.67 ± 48.97. The results from the two-way mixed factorial ANOVA indicated that there was a significant time × group interaction (*F* = 24.259, *p* < 0.001, η_p_^2^ = 0.464). The results from the Bonferroni corrected pairwise comparisons showed significant increases in leg press 5RM for the traditional group (*p* < 0.001) and functional group (*p* = 0.006). However, the magnitude of improvement was >3× greater for the traditional group (*d* = 1.20 *versus d* = 0.385). The number of participants that exceeded the MID of 18.54 kg in the traditional and functional groups was 12 (100%) and 11 (16.1%), respectively.

### Leg extension 5RM

The pretest and posttest mean ± SD leg extension 5RM values (kg) for the traditional group were 30.95 ± 14.66 and 43.11 ± 14.26, respectively. These corresponding values for the functional group were 32.48 ± 14.81 and 38.64 ± 18.30. The results from the two-way mixed factorial ANOVA indicated that there was no significant time × group interaction (*F* = 3.554, *p* = 0.070, η_p_^2^ = 0.113), as well as no main effect for group (*F* = 0.067, *p* = 0.798, η_p_^2^ = 0.542). There was, however, a significant main effect for time (*F* = 33.180, *p* < 0.001, η_p_^2^ = 0.542). The results from the Bonferroni corrected pairwise comparison indicated that, when collapsed across group, leg extension 5RM increased from 31.7 ± 2.7 to 40.9 ± 3.1 kg. While the interaction term was not statistically significant, the pre-post effect size for the traditional group was medium/large (*d* = 0.768), whereas the improvement for the functional group was small (*d* = 0.389). The number of participants that exceeded the MID of 6.94 kg in the traditional and functional groups was 9 (75.0%) and 5 (27.8%), respectively.

### Leg curl 5RM

The pretest and posttest mean ± SD leg curl 5RM values (kg) for the traditional group were 18.18 ± 9.54 and 21.10 ± 9.41, respectively. These corresponding values for the functional group were 19.03 ± 10.39 and 21.02 ± 10.76. The results from the two-way mixed factorial ANOVA indicated that there was no significant time × group interaction (*F* = 0.786, *p* = 0.383, η_p_^2^ = 0.027), as well as no main effect for group (*F* = 0.011, *p* = 0.919, η_p_^2^ < 0.001). There was, however, a significant main effect for time (*F* = 22.376, *p* < 0.001, η_p_^2^ = 0.444). The results from the Bonferroni corrected pairwise comparison indicated that, when collapsed across group, leg curl 5RM increased from 18.6 ± 1.9 to 21.1 ± 1.9 kg. The number of participants that exceeded the MID of 4.64 kg in the traditional and functional groups was 2 (16.7%) and 1 (5.56%), respectively.

### VL CSA

**FIGURE 6:**
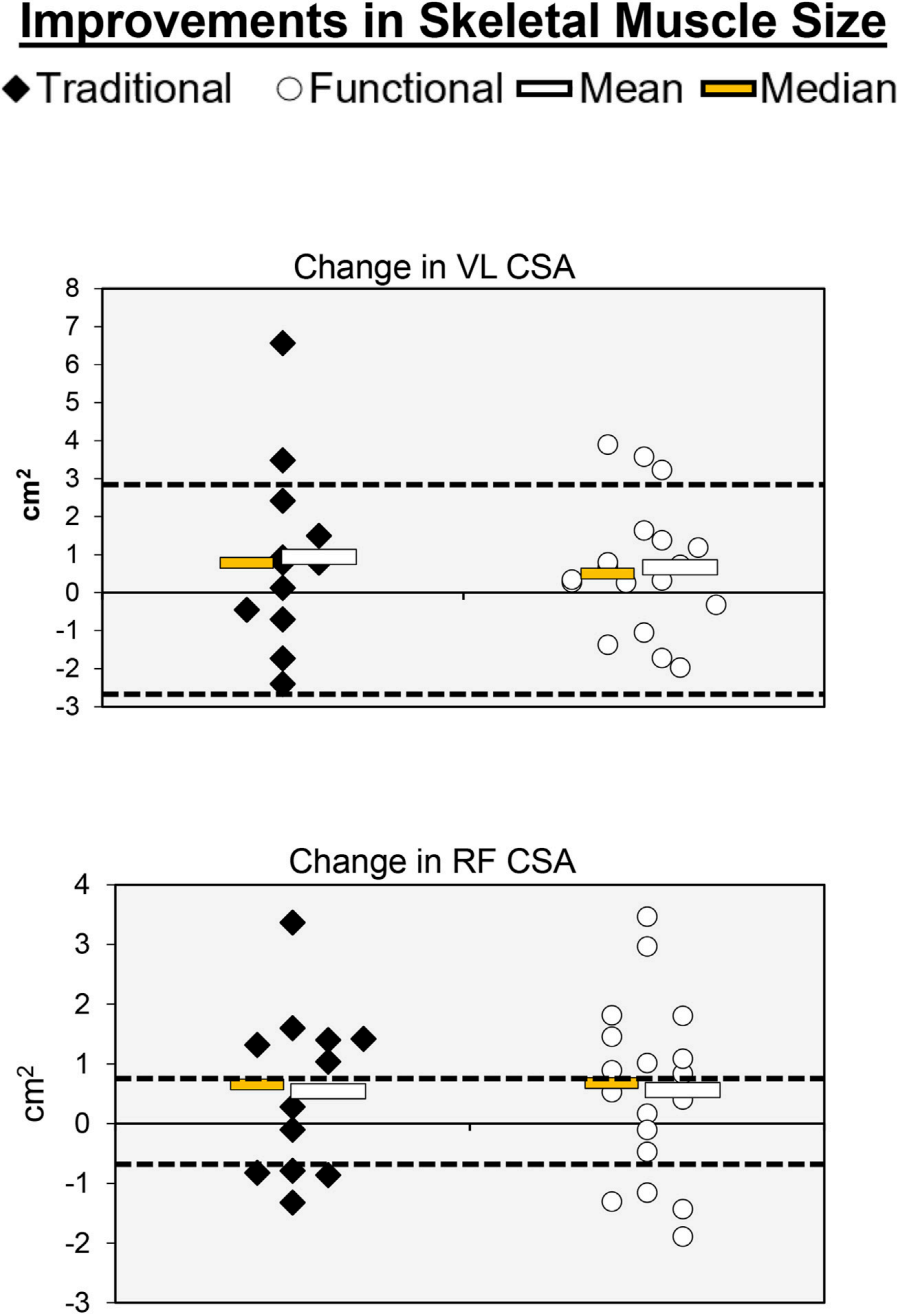
Individual participant, mean, and median changes for RF CSA and VL CSA for both training groups. The dotted red lines represent the MID.

Individual participant and mean changes for CSA have been displayed in Figure 6. The pretest and posttest mean ± SD VL CSA values (cm^2^) for the traditional group were 14.53 ± 5.06 and 15.46 ± 4.65, respectively. These corresponding values for the functional group were 14.96 ± 4.05 and 15.62 ± 4.65. The results from the two-way mixed factorial ANOVA indicated that there was no significant time × group interaction (*F* = 0.131, *p* = 0.720, η_p_^2^ = 0.005), as well as no main effect for group (*F* = 0.031, *p* = 0.861, η_p_^2^ = 0.001). However, there was a significant main effect for time (*F* = 4.529, *p* = 0.042, η_p_^2^ = 0.139). The number of participants that exceeded the MID of 2.77 cm^2^ in the traditional and functional groups was 2 (16.7%) and 3 (16.7%), respectively.

### RF CSA

The pretest and posttest mean ± SD RF CSA values (cm^2^) for the traditional group were 4.11 ± 1.68 and 4.66 ± 2.24, respectively. These corresponding values for the functional group were 4.18 ± 1.47 and 4.74 ± 1.61. The results from the two-way mixed factorial ANOVA indicated that there was no significant time × group interaction (*F* = 0.001, *p* = 0.973, η_p_^2^ < 0.001), as well as no main effect for group (*F* = 0.016, *p* = 0.902, η_p_^2^ < 0.001). There was, however, a significant main effect for time (*F* = 4.301, *p* = 0.047, η_p_^2^ = 0.133). The results from the Bonferroni corrected pairwise comparisons indicated that, when collapsed across group, RF CSA increased from 4.15 ± 0.29 to 4.70 ± 0.35 cm^2^. The number of participants that exceeded the MID of 0.84 cm^2^ in the traditional and functional groups was 8 (66.7%) and 13 (72.2%), respectively. One of the participants that exceeded the MID in the functional group showed a decrease in RF CSA.

### Physical activity and nutrition

For step counts and the percentage of time spent in sedentary and vigorous activity, the results indicated that there was no significant time × group interactions (*F* < 0.798, *p* > 0.380, η_p_^2^ < 0.029) and no main effects for group (*F* < 1.703, *p* > 0.203, η_p_^2^ < 0.059) or time (*F* < 2.161, *p* > 0.153, η_p_^2^ < 0.074). There was also no significant time × group interactions (*F* < 0.505, *p* > 0.483, η_p_^2^ < 0.018) or main effects for group (*F* < 1.703, *p* > 0.203, η_p_^2^ < 0.059) for the percentage of moderate activity and percent of moderate-to-vigorous physical activity (MVPA). However, there were significant main effects for time in percentage of moderate activity (*F* = 5.770, *p* = 0.023, η_p_^2^ = 0.176) and percent of MVPA (*F* = 5.456, *p* = 0.027, η_p_^2^ = 0.168). The marginal means (mean ± SE) for time in percentage of moderate activity and percent of MVPA was as follows: % of MOD PRE = 4.7 ± 0.0; % of MOD POST = 8.6 ± 0.0; % MVPA PRE = 4.7 ± 1.5; % MVPA POST = 8.5 ± 1.5.

For the nutrition analyses, the results indicated that there was no significant time × group interactions (*F* < 1.521, *p* > 0.228, η_p_^2^ < 0.052), as well as no main effects for time (*F* < 1.432, *p* > 0.241, η_p_^2^ < 0.049) or group (*F* < 0.259, *p* > 0.615, η_p_^2^ < 0.009). When collapsed across time, the mean ± SE nutrition data was as follows: kcal: traditional = 1593.2 ± 117.4 vs. functional = 1520.52 ± 95.89; protein (grams): traditional = 82.3 ± 4.6 vs. functional = 79.3 ± 3.7; protein (grams×kg^-1^): traditional = 1.20 ± 0.10 vs. functional 1.13 ± 0.08; carbohydrate (grams): traditional = 147.4 ± 19.2 vs. functional = 156.3 ± 15.7; fat (grams): traditional = 67.9 ± 7.4 vs. functional = 67.7 ± 6.0.

### Correlation between change scores

**FIGURE 7:**
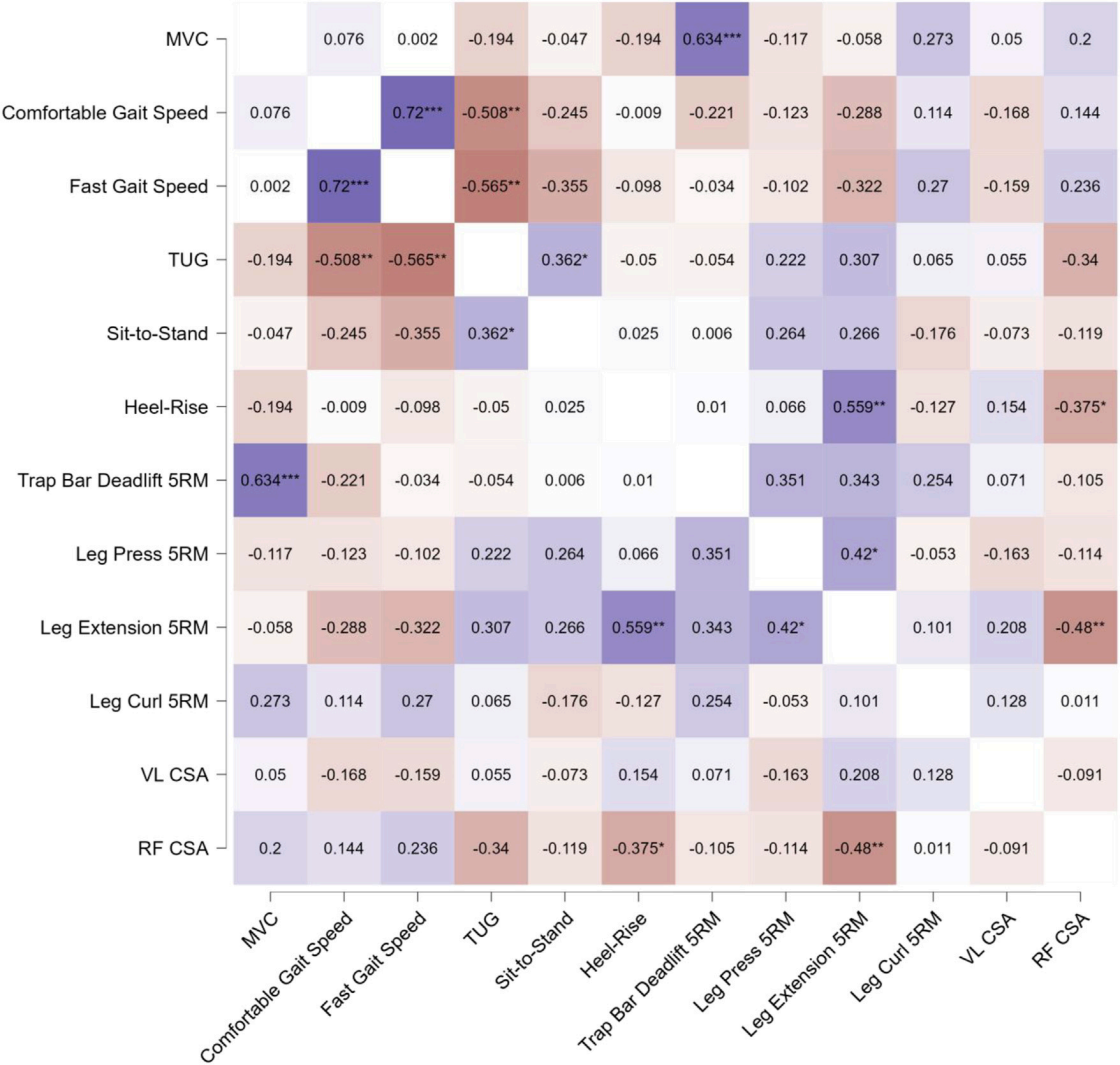
Pearson’s product-moment correlation coefficient (*r*) heatmap visualizing the relationships between pretest-posttest change scores for the assessed variables across the entire participant sample, without consideration of group assignment. The color scale represents the magnitude and direction of the correlations, with positive correlations shown in shades of red, negative correlations in shades of blue, and the intensity of the color corresponding to the strength of the relationship. The diagonal elements display the correlation of each variable with itself (*r* = 1.0), while the off-diagonal elements illustrate the pairwise correlations between the different variables. Statistical significance of the correlations is indicated by asterisks, where * denotes *p* < 0.05, ** denotes *p* < 0.01, and *** denotes *p* < 0.001. This comprehensive heatmap visualization allows for a quick assessment of the pattern and strength of relationships between the pretest-posttest changes in the assessed physical function and muscle morphology measures across the entire participant sample.

Figure 7 summarizes the results from our 66 Pearson *r* correlation analyses. Overall, we observed relatively few (9/66; 13.6%) significant associations between changes in our dependent variables. Across the entire sample, the most noteworthy correlations were between MVC force and Trap Bar Deadlift 5RM (*r* = 0.634, *p* < .001), comfortable and fast gait speed (*r* = 0.720, *p* < .001), comfortable gait speed and TUG (*r* = −0.508, *p* = .004, fast gait speed and TUG (*r* = −0.565, *p* = .001, TUG and sit-to-stand (*r* = 0.362, *p* = .049), heel-rise and leg extension 5RM (*r* = .559, *p* = .001), heel-rise and RF CSA (*r* = −.375, *p* = .041), leg press and leg extension 5RM (*r* = .420, *p* = .021), and leg extension 5RM and RF CSA (*r* = .480, *p* = .007).

## Discussion

Resistance training is a potent stimulus for improving muscle strength and size among older adults (Chodzko-Zajko et al., 2009; Westcott, 2012; Fragala et al., 2019). Much of the resistance training literature (American College of Sports Medicine, 2009; Sheppard and Triplett, 2016) and subsequent guidelines from leading organizations (ACSM and NSCA) focus on exercises that require free-weights and machines. However, assessments that are used in the screening and diagnosis of sarcopenia typically involve functional, everyday movements, such as walking and standing from a chair (Cruz-Jentoft et al., 2014; 2019). The present study tested the hypothesis that resistance training adaptations exhibit task-specificity among older adults. Our key findings were largely consistent with our hypothesis. Older adults that performed traditional resistance training showed significant improvements in 5RM strength that had only limited carryover to functional assessments commonly used to screen for and diagnose sarcopenia such as gait-speed and chair stands. In contrast, participants that performed functional resistance training exercises with a weighted vest showed notable task-specific improvements in functional measures, but these adaptations had smaller carryover to 5RM strength. Our work also demonstrated that both types of training improved strength in a non-specific task (isometric knee extensor force) and muscle size, suggesting that some aspects of observed adaptations were generic. These results have important implications for exercise prescription, suggesting that clinicians should utilize targeted functional or traditional exercises to optimize specific outcomes in older adults. Below we discuss the implications, impact, strengths, and limitations associated with these findings.

Previous studies in younger adults have highlighted the importance of task-specificity for improving maximal strength. Perhaps the most influential study was published by Rutherford and Jones (1986). Their work suggested that a significant portion of the initial strength gains observed after a brief period of resistance training were attributed to neural adaptations, including improved motor learning and coordination. In that study, unilateral concentric knee extension training resulted in nearly a 200% increase in the loads used during training, but this resulted in only a 15%–20% increase in isometric force. Similar findings were more recently reported by Mattocks et al. (2017), who demonstrated that practicing 1RM tests resulted in similar outcomes as participating in a conventional resistance training program. While these studies have helped solidify the concept of task-specificity, less direct attention has been given to task-specificity for older adults hoping to maintain physical function throughout aging. A study by Helbostad et al. (2004) compared the effectiveness of a home training group (TUG, sit-to-stand, fast and preferred gait speed, etc.) and a combined training group (group exercise and home exercise) on functional performance in older adults. They found that older adults in the home training group showed increases in functional performance similar to those of the combined group. Tou et al. (2021) reported similar improvements in functional performance among frail community-dwelling older adults when undergoing a 12-week functional performance training program compared to a control group. Utilizing a randomized controlled trial, Manini et al. (2005) investigated the effects of resistance, functional, and combined training on knee extensor steadiness in older adults with functional limitations. Their findings indicated that resistance training reduced force fluctuations during short, fast contractions, while functional training decreased sway during standing. Manini et al. (2005) concluded that targeted resistance and functional exercises can improve neuromuscular control in a task-specific manner, suggesting their value in enhancing steadiness and reducing fall risk in functionally limited older adults. Our results extend these concepts and provide greater support for the concept of task-specificity in older adults.

It is important to highlight the unique aspect of using a weighted vest for the functional training program implemented in the present study. Acknowledging the importance of the overload principle for older adults (Devries and Giangregorio, 2023), we utilized a weighted vest to allow for greater physiological stress and adaptation starting as low as 5%–10% body mass and increased the load incrementally based on individual participant responses as the study progressed. This accessible approach resulted in task-specific adaptations and has high feasibility for unsupervised home programs. Research studies that have utilized weighted vests during resistance training in older adults have consistently shown benefits *versus* non-vested training, with significant improvements in lower extremity strength, power, and balance (Bautmans et al., 2005; Straight et al., 2015). Weighed vest training also improves mobility, dynamic balance, and stair climbing power compared to standard training (LaStayo et al., 2003). In addition, wearing a weighted vest for 8 hours per day for 3 weeks, independent of specific exercise training, has been shown to improve body composition among mildly obese participants (Ohlsson et al., 2020). We believe that our findings are particularly important because while the functional training performed in our study was performed under supervised laboratory conditions, weighted vest training can easily be implemented by older adults at home. The utilization of a weighted vest may help overcome many of the commonly reported barriers to resistance training among older adults, such as a lack of transportation, fear of injury, and perceptions that the use of free weights and machines are designed for younger adults (Sims et al., 2010; Burton et al., 2015). It is our hope that our results provide support for additional weighted vest training research among older adults, particularly in helping to overcome barriers and encouraging participation in resistance training. As we did not evaluate perceptions of the two types of resistance training implemented in the present study, future research is needed to examine the extent to which older adults enjoy and adhere to functional resistance training with a weighted vest. Furthermore, the use of a weighted vest should be carefully considered in clinical populations with neurologic injury, balance issues, and a history of falls. With additional supporting research, this simple and scalable exercise intervention could potentially help combat sarcopenia and preserve independence in older adults.

In addition to our assessment of improvements in task-specific performance, we also evaluated whether traditional and functional resistance training would result in more generic muscular adaptations. Our findings demonstrated that both training programs resulted in significant increases in isometric MVC force and CSA of the VL and RF muscles. While both resistance training regimens required activation of the knee extensors, neither included isometric contractions specifically. This suggests that, within each intervention group, participants exhibited enhancements in task-specific performance, isometric MVC force, and muscle hypertrophy, but potentially smaller changes in non-specific neuromuscular performance. In agreement with the present results, recent investigations have illustrated that functional exercises mimicking activities of daily living such as chair stands, walking, and balance training can stimulate muscle growth in older adults (Hortobágyi et al., 2015; Gadelha et al., 2018; Lizama-Pérez et al., 2023). For example, Lizama-Pérez et al. (2023) found that 12 weeks of chair stand training increased thigh muscle CSA and strength in older females. Similarly, Hortobágyi et al. (2015) reported that 6 weeks of bodyweight-supported treadmill training led to greater quadriceps muscle volume in frail elders. Gadelha et al. (2018) also showed that balance training over 6 months increased soleus and tibialis anterior muscle size by 5%–8% in adults ≥65 years of age. Collectively, our findings contribute to a growing body of literature demonstrating that progressive, functional exercises can provide a sufficient hypertrophic stimulus to counteract sarcopenia.

While our hypothesis was generally supported by the data, some aspects require clarification and nuance. First, heel rise performance, which depends on plantarflexor strength and power, showed a modest, non-significant improvement (main effect for time *p* = 0.052). A potential explanation is that heel rise training in the functional group was performed at the end of each training session when participants were fatigued. It is also possible that improvements in heel rise performance require greater loads or higher training frequencies. Furthermore, we observed improvements in chair sit-to-stand performance and 5RM knee curl strength, but no differences between groups. These findings suggest that the functional training was particularly helpful for improving mobility tasks involving walking, but not those without a walking component. Given the similar improvements in isometric strength and CSA, it seems that both training programs produced similar intramuscular effects. However, they differed in neuromuscular control of different muscle groups, which is specific to the training type. From a research perspective, understanding the mechanistic underpinnings of these findings warrants further investigation. Additionally, clinicians working with older adults in need of plantarflexion strength might consider higher volumes of targeted training, as the training programs implemented in this study were not sufficient for improving heel rise performance.

Three additional details related to our study design and results that should be deliberated include our analysis of 6 days of dietary food logs (four weekdays, two weekend days), the assessment of unstructured physical activity levels outside of the study, and our inclusion of the MID to assess individual participant responses. While we did not observe significant or meaningful changes over time or differences in caloric or macronutrient consumption between groups, the protein consumption of our older adult participants (mean = 80.8 g) was likely too low to maximize anabolism and training adaptations (Bauer et al., 2013). The protein needs of older adults participating in resistance training has received significant attention in recent years, and while recommendations for daily consumption may vary depending on a variety of factors, it is generally accepted that the protein and essential amino acid needs of older adults are greater than those for younger adults (Baum et al., 2016). Thus, the improvements in muscle strength and size reported herein may have occurred despite insufficient protein intake, suggesting that further improvements may be possible with even greater protein consumption. Another key finding of this study was that participation in both resistance training programs resulted in significant increases in moderate and MVPA among the older adult participants during the final week compared to baseline, as assessed objectively via accelerometry. These changes were observed irrespective of total step counts. The improvements in muscular strength and size observed following the resistance training programs may have been influenced by this increase in higher intensity ambulatory activity. However, we also interpret the increase in moderate and MVPA as a positive alteration in physical activity behavior potentially driven by study participation. The resistance training programs may have provided the older adults with heightened capability, independence, and confidence to engage in more intense activities, like jogging rather than just walking. Further research is warranted to elucidate the potential bidirectional relationship between resistance training-induced muscular adaptations and concurrent changes in physical activity patterns among older adults. Lastly, the results from our group mean changes were largely corroborated by individual participant responses, which were evaluated by the MID statistic as originally described by Norman et al. (2003). It is important to note that the MID statistic used in our study differs from other clinical metrics such as the minimal clinically important difference and other cut-points developed specifically for research involving older adults. While these alternative approaches offer certain advantages, our study design enabled the calculation of sample-specific reliability and change assessments tailored to our population. Given the relatively high functional status of our study participants, metrics designed for sarcopenic individuals may not be appropriate for our sample. Therefore, we chose to utilize the MID as it provided a more relevant and contextualized interpretation of the observed changes within our specific cohort.

Our study had several strengths that should be taken into consideration when placing the findings in context. First, our study was bolstered by a rigorous experimental design with multiple controls (e.g., consistent time of day and laboratory staff supervision, concern for nutrition, physical activity, and hydration). Our participants were invested in the study and attended 100% of their scheduled testing and training sessions. In addition, all participants were thoroughly familiarized with the tests and exercises, and two separate pretest sessions were used to establish unique, sample-specific reliability statistics, thereby allowing us to report the extent to which each participant’s change was meaningful. We also elected to study a training frequency of twice/week to reflect physical activity guidelines for older adults (CDC, 2023). However, our work had several limitations as well. First, our study sample was generally healthy, independent of major physical limitations, and community-dwelling. Thus, the extent to which these findings can be directly applied to frail or sarcopenic older adults is unknown. Second, we did not study upper-body muscles, nor did we include other physical activity interventions that would have resulted in more comprehensive, health promoting adaptations (e.g., aerobic exercise or flexibility training). At the conclusion of our study, some participants commented that they would have liked to engage in upper-body resistance training. Third, we acknowledge that our protocol to initially load and subsequently overload the weighted vest was not entirely objective, in contrast to the well-established 5RM strength testing for the traditional group. It is therefore possible that the relative weight of the vest varied across participants. While this may be perceived as a limitation, we believe that our approach likely reflects what an older adult may do with a weighted vest under unsupervised conditions at home. Similarly, the traditional resistance training program used in the present study was designed to enhance 5RM strength. Additional research would be needed to determine if manipulation of various resistance training program variables (e.g., heavier/lighter loads, longer/shorter rest intervals, greater training frequency, etc.) would elicit different results. It was also beyond the scope of our study to include outcome measures that would have provided insights into neuromuscular adaptations during the early phase of the training programs (e.g., surface electromyography, evoked potentials, transcranial magnetic stimulation). Thus, while we suspect that neuromuscular factors facilitated task-specific adaptations, our included dependent variables did not allow for these analyses. Finally, our laboratory staff and investigators were not blinded to group assignment during testing. These strengths and limitations are important to consider but represent exciting opportunities for additional older adult research.

In summary, the principle of training specificity should be a cornerstone of exercise programming for older adults. While traditional resistance training has numerous benefits, our findings suggest that clinicians can also prescribe targeted functional exercises that mimic daily activities to optimize physical performance and mobility outcomes in older adults. Functional training elicits task-specific adaptations that may better enhance mobility and independence *versus* traditional resistance training programs. It was also noted that concurrent hypertrophic and isometric strength changes imply that task-specific adaptations occurred alongside more generic muscular responses. Further research should explore the feasibility and adherence of unsupervised functional resistance training to support long-term engagement. Overall, this study exemplifies the importance of tailored, functional resistance exercise for combating sarcopenia and preserving strength and autonomy in an aging population.

## Data Availability

The raw data supporting the conclusion of this article will be made available by the authors, without undue reservation.
